# T1 and T2 mapping detect myocardial edema after repeated 200J electrical cardioversion

**DOI:** 10.1186/1532-429X-16-S1-P157

**Published:** 2014-01-16

**Authors:** Dominik P Guensch, Janelle Yu, Kady Fischer, Gobinath Nadeshalingam, Matthias G Friedrich

**Affiliations:** 1 Philippa &Marvin Carsley CMR Centre, Montreal Heart Institute, Montreal, Quebec, Canada; 2 Anesthesiology and Pain Medicine, University Hospital Bern, Bern, Switzerland

## Background

Electrical defibrillation and cardioversion are important and sometimes life-saving interventions. However, it remains controversial whether the intervention itself can significantly damage the heart. Myocardial edema is an early feature of myocardial injury that can be visualized by T2-weighted sequences. Newer approaches utilize T2 as well as T1 mapping techniques. We used T1 and T2 mapping to detect edema as a marker for defibrillation injury.

## Methods

Using a 3T clinical MRI system, we assessed 10 anesthetized pigs at baseline and hourly for 5 hours after 5 consecutive synchronized transthoracic shocks of 200J and acquired images for T1 maps (MOLLI, modified look-locker inversion recovery) and T2 maps in three short-axis planes; basal, mid and apical. Six pigs undergoing the same protocol yet without shocks served as controls. After euthanasia and explantation of the hearts, tissue samples were obtained from regions of interest (ROI) defined by positive CMR results. Hematoxylin and eosin (HE) stains of these samples were then assessed for intercellular and interstitial edema using randomly sampled planimetry.

## Results

In all 10 pigs undergoing shocks, we observed myocardial injury, which was located in the anterior and inferior wall (n = 8), septal and inferior segments (n = 1) or septal and lateral segments (n = 1). In T1 and T2 maps, myocardial edema was predominantly observed in the basal slices. T2 was significantly increased from baseline at the first hour and at hours 3-5 by up to 4.2 ± 0.9% (p < 0.05, Figure 2) on a whole heart analysis, while T2 dropped in the remote myocardium (max. -2.6 ± 1.2%, n.s.) and in the control pigs (max. -6.1 ± 0.7%, p = 0.02). On a slice by slice analysis the maximal increase was 11.3 ± 3.9% in the basal slice (p < 0.05). T1 was increased in affected ROI at 3 and 4 h post shock (3.8 ± 0.4% and 5.0 ± 0.3% p < 0.01). Histology data showed that myocardial cell area increased by 185.65% (p = 0.016) while interstitial area was expanded 953.06% compared to healthy control myocardium (p = 0.016) in regions being deemed injured by either T1 or T2 maps. Both, cell area and interstitial space were correlated to T1 (ms) in ROI (r = 0.6/p = 0.04 and r = 0.44/p = 0.037, respectively).

**Figure 1:**
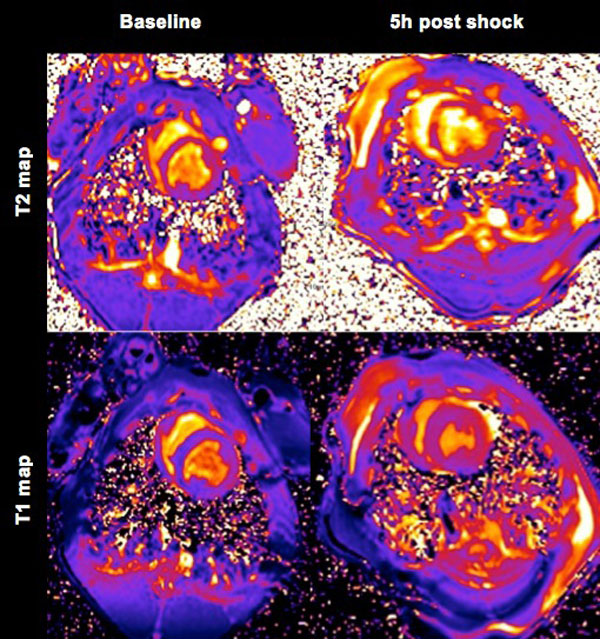
**T1 and T2 maps at baseline before (left) and 5 h after cardioversion (right) of 5 × 200J, The T2 maps show a T2 increase in the right pectoral muscle, the anterior RV and the septum**. The T1 maps exhibit the location of the defibrillation pads and global T1 increase in the LV.

**Figure 2:**
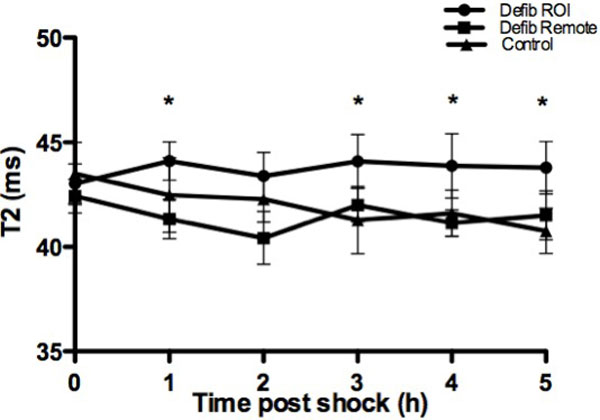
Mean ± SEM change of T2 in regions of interest (ROI), remote myocardium of defibrillated pigs and in the myocardium of control pigs in the whole heart based analysis (*p < 0.05 ROI vs. Remote).

## Conclusions

Repeated cardioversion/defibrillation consistently leads to cellular and interstitial myocardial and skeletal edema, which can be visualized by T1 mapping and T2 mapping.

## Funding

Funding is provided by the Montreal Heart Institute Foundation and the Canadian Foundation for Innovation.

